# Design, Synthesis, and Molecular Docking of Triazole‐Coumarin Hybrids as Potent Breast Cancer Inhibitors Targeting Cell Cycle and Apoptosis

**DOI:** 10.1002/cbdv.202501775

**Published:** 2025-09-05

**Authors:** Murat Keser, Emre Menteşe, Suleyman Ilhan, Mustafa Emirik, Harika Atmaca

**Affiliations:** ^1^ Department of Medical Oncology, Izmir Tepecik Education and Research Hospital University of Health Sciences Izmir Türkiye; ^2^ Department of Chemistry, Faculty of Science and Art Recep Tayyip Erdogan University Rize Türkiye; ^3^ Department of Biology, Faculty of Engineering and Natural Sciences Manisa Celal Bayar University Manisa Türkiye

**Keywords:** apoptosis, breast cancer, cell cycle, molecular docking, triazole‐coumarin hybrid

## Abstract

Breast cancer continues to pose a significant global health burden, highlighting the urgent need for novel chemotherapeutic agents with improved selectivity and reduced toxicity. In this study, we rationally designed and synthesized six novel amide‐bridged triazole‐coumarin hybrids (**5a–f**) based on the known anticancer potential of both pharmacophores. The synthesized compounds were evaluated for their cytotoxicity in MCF‐7 and MDA─MB─231 breast cancer cell lines and non‐tumorigenic MCF‐10A cells. Among them, derivative **5f** showed the most potent anticancer activity with minimal toxicity toward normal cells. Mechanistic studies revealed that **5f** induced apoptosis by modulating Bax and Bcl─2 expression and arrested the cell cycle at the S phase via downregulation of CDK2 and Cyclin E. Molecular docking analyses confirmed its high binding affinity to CDK2 and Bcl─2, supporting its potential as a dual‐target inhibitor. These findings suggest that compound **5f** is a promising lead structure for the development of selective anticancer agents targeting breast cancer.

## Graphical abstract

Triazole‐coumarin hybrids were synthesized as breast cancer inhibitors. Derivative **5f** induced S‐phase arrest and apoptosis in cancer cells, with minimal toxicity to healthy cells, showing promise for anticancer drug development.

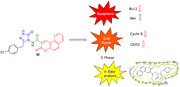

## 1 Introduction

Breast cancer remains the most commonly diagnosed cancer and the leading cause of cancer‐related death among women worldwide. According to GLOBOCAN 2022 estimates, breast cancer was the most commonly diagnosed cancer worldwide, with approximately 2.3 million new cases and 685 000 deaths reported in 2020, highlighting the urgent need for safer and more effective therapeutic strategies [1]. Despite advances in diagnosis and treatment, the need remains for novel chemotherapeutic agents that offer high efficacy and minimal side effects, particularly for aggressive breast cancer subtypes.

One promising strategy is molecular hybridization, which involves the combination of two or more bioactive scaffolds into a single molecule to enhance biological activity and overcome limitations of single‐target agents. Coumarins (2H‐1‐benzopyran‐2‐one) are phenolic compounds that belong to the benzopyrone class, characterized by fused benzene and pyrone rings [2]. Coumarins and their derivatives possess a broad spectrum of therapeutic properties, including antimicrobial, cancer‐fighting, HIV‐inhibiting, anti‐inflammatory, anticoagulant, antimalarial, and free radical‐scavenging effects [2, 3]. These derivatives can interact with various cellular molecules, including kinases, telomerase, aromatase, sulfatase, monocarboxylate transporters, and carbonic anhydrase. As a result, they can induce cell cycle arrest, promote cell death, inhibit angiogenesis, suppress kinase and telomerase activity, exhibit antimitotic effects, and block monocarboxylate transporters. These broad‐spectrum properties indicate that coumarin is a superior part of the generation of innovative anticancer medications [3, 4, 5].

Numerous hybrid compounds, including cefatrizin, AT‐3639, voreloxin, and quarfloxin, are currently used in the treatment of cancers, and some of them are continuing clinical studies [5, 6]. To provide novel anticancer candidates, it seems reasonable that the coumarin moiety may be hybridized with other anticancer pharmacophores. With this process, it is possible to increase therapeutic activity and affinity while lowering adverse effects and overcoming drug resistance [5].

Triazoles are chemical structures formed by a five‐membered ring that includes three nitrogen atoms and two carbon atoms in its composition. Their derivatives have garnered attention as crucial building blocks in the development of innovative anti‐cancer treatments due to their remarkable efficacy and minimal toxicity [7, 8]. Importantly, several triazole‐based molecules have demonstrated the ability to induce apoptosis and arrest the cell cycle by modulating key regulatory proteins such as caspases, cyclin‐dependent kinases (CDKs), and Bcl─2 family members [9, 10, 11, 12]. Thus, both coumarin and triazole scaffolds provide complementary anticancer mechanisms, making their hybridization a rational approach for drug development. The integration of coumarin and triazole moieties into a single hybrid structure has previously yielded compounds with enhanced bioactivity. However, amide‐bridged triazole–coumarin hybrids remain poorly explored, especially in the context of breast cancer, and their full therapeutic potential and mechanism of action are yet to be elucidated.

Molecular modeling and docking studies are powerful tools widely used in drug design to estimate the interactions of compounds with biological targets. These methods give a clearer insight into the biological activities of new molecules and are valuable for explaining the interaction modes of potential inhibitors [13].

In this study, we aimed to design and synthesize a novel series of triazole–coumarin hybrids (**5a–f**) linked via an amide bridge, based on the hypothesis that this structural motif could yield dual‐target inhibitors capable of inducing apoptosis and arresting the cell cycle in breast cancer cells. We systematically evaluated their cytotoxicity, apoptotic, and cell cycle effects in MCF‐7 and MDA‐MB‐231 cell lines, and further explored their binding affinity to CDK2 and Bcl─2 through molecular docking studies. These findings offer new insight into the potential of these hybrids as selective and potent breast cancer therapeutics.

## 2 Results and Discussion

### 2.1 Chemistry

**SCHEME 1:**
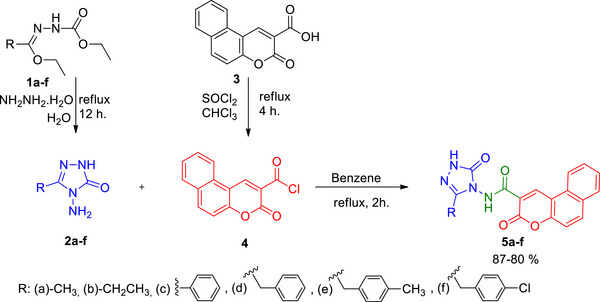
Synthetic route of compounds **5a–f**.

The amide‐bridged triazole‐coumarin hybrids (**5a–f**) were synthesized following the methods described in Scheme 1. Compounds 1a‐f and compound **3** were prepared based on procedures from previously published literature [14, 15].

The first intermediates (**2a–f**) were reached by the reaction of **1a‐f** with hydrazine monohydrate [14]. 3‐Oxo‐3*H*‐benzo[*f*]chromene‐2‐carbonyl chloride (**3**), which is the second intermediate, was synthesized by the reaction of 3‐oxo‐3*H*‐benzo[*f*]chromene‐2‐carboxylic acid (**2**) and SOCl_2_ in chloroform under reflux [15]. Compounds **2a‐f** were reacted with compound **4** to obtain triazole‐coumarin hybrids (**5a–f**) containing an amide bridge.

**SCHEME 2:**
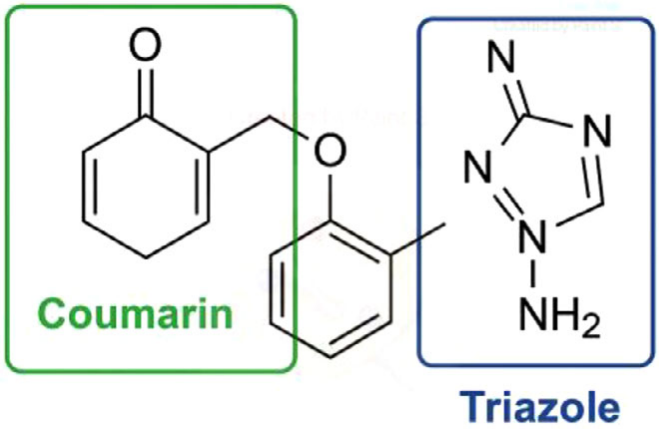
Representative chemical structure of a triazole–coumarin hybrid molecule, highlighting the coumarin and triazole heterocyclic moieties.

To better illustrate the structural features of the synthesized compounds, a representative structure highlighting the triazole and coumarin rings is presented in Scheme 2, where the key heterocyclic pharmacophores are marked.

### 2.2 Biological Evaluation

#### 2.2.1 Cytotoxic Activity of Synthesized Triazole‐Coumarin Hybrids

Among the primary goals of medicinal and organic chemistry is the synthesis and creation of molecules that have therapeutic effects. For this purpose, attempts are made to create new therapeutic molecules by hybridizing pharmacophores known to have strong cytotoxic effects and few side effects. These unique pharmacophores have a variety of modes of action in addition to selectivity. Coumarin hybrids have recently gained the intense interest of researchers to investigate their effectiveness as a treatment against breast cancer due to their minimal toxicity profile. By associating itself with several biological targets associated with breast cancer, coumarin hybrids have been shown to have strong anti‐breast cancer activity.

Here, we examined the cytotoxic effects of recently synthesized triazole‐coumarin hybrids on healthy MCF‐10A cells as well as two distinct breast cancer cells, MDA‐MB‐231 and MCF‐7. All the synthesized derivatives showed time‐ and concentration‐dependent cytotoxic activity against both breast cancer cells. However, the highest cytotoxicity was detected at 72 h, and thus, 50% inhibition concentration (IC_50_) values were measured at 72 h. The IC_50_ of synthesized triazole‐coumarin hybrid derivatives (**5a–f**) and reference drug cisplatin in all tested cells are presented in Table 1.

**TABLE 1:** Inhibition concentration 50% (IC_50_) values of synthesized triazole‐coumarin hybrid derivatives (**5a–f**) and reference drug cisplatin in MCF‐10A human breast epithelial cells, MCF‐7, and MDA‐MB‐231 breast cancer cells (µM).

Derivative	MCF‐10A	MDA‐MB‐231	MCF‐7
**5a**	12.5± 1.0	18.4 ± 1.16	22.5 ± 1.8
**5b**	25.2± 1.5	95.8 ± 2.4	76.6 ± 2.1
**5c**	62.8 ± 0.4	64.0 ± 1.4	25.9 ± 0.8
**5d**	38.2 ± 0.7	87.0 ± 2.0	49.8 ± 2.2
**5e**	40.6± 2.1	61.7 ± 0.4	68.9 ± 3.1
**5f**	**61.9± 0.6**	**15.7 ± 0.2**	**22.1 ± 0.42**
**Cisplatin**	12.2 ± 0.8	8.7 ± 0.6	9.3 ± 1.2

**FIGURE 1:**
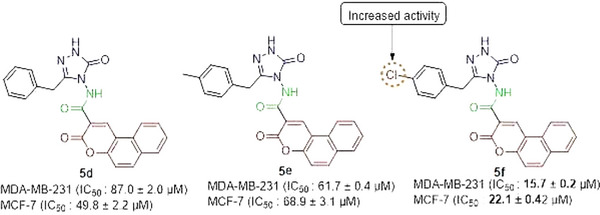
Structure‐activity relationship of compounds **5d**, **5e**, and **5f** against breast cancer cell lines. Chemical structures of three synthesized compounds (**5d**, **5e**, and **5f**) are shown, with key functional groups highlighted. IC_50_ values (mean ± SD) for MDA‐MB‐231 and MCF‐7 breast cancer cell lines indicate cytotoxic activity. The introduction of a chlorine (Cl) substituent in compound **5f** significantly enhances anticancer activity, as indicated by the lower IC_50_ values compared to **6d** and **6e**.

Among the synthesized derivatives, compound **5f,** containing a chlorine atom, was the most cytotoxic compound with an IC_50_ value of < 25 µM in both cancerous cells (Figure 1). Chlorine atoms have a positive effect on activity. This result is also consistent with our previous study, in which coumarin‐triazole hybrid compounds were synthesized as potential anticancer agents.

Although some of the synthesized compounds exceed 500 Da in molecular weight (MW), this does not necessarily limit their potential as drug candidates. Many clinically approved anticancer agents, such as paclitaxel (MW ∼854 Da) and eribulin (MW ∼729 Da), fall outside the traditional “Rule of Five” yet demonstrate excellent biological activity. The observed in vitro cytotoxicity of our compounds suggests sufficient membrane permeability and cellular uptake, likely facilitated by their favorable lipophilicity and structural features. Therefore, while MW is an important consideration, it should be interpreted alongside other pharmacokinetic and physicochemical properties that contribute to drug‐likeness.

**FIGURE 2:**
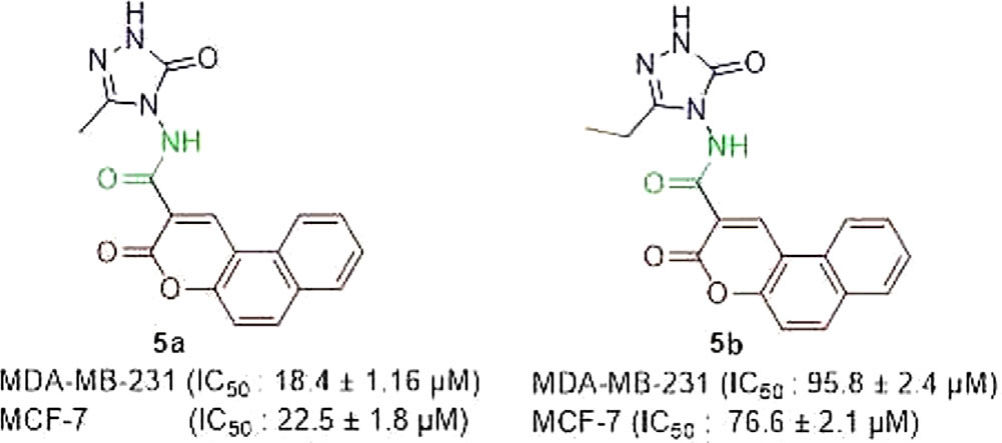
Comparison of the cytotoxic effects of compounds **5a** and **5b** against breast cancer cell lines. Chemical structures of compounds **5a** and **5b** are displayed, highlighting their structural differences. IC_50_ values (mean ± SD) for MDA‐MB‐231 and MCF‐7 breast cancer cell lines indicate that **5a** exhibits significantly higher cytotoxic activity compared to **5b**. The observed difference suggests that the ethyl substitution in **5b** reduces anticancer potency.

In the literature, hybrids of coumarin‐1,2,3‐triazole‐dithiocarbamate were examined, and how they affected different human cancer cells. Strong anticancer activity resulted from the replacement of the coumarin moiety at position 4 with a 1,2,3‐triazole‐dithiocarbamate residue. IC_50_ value of the derivative on MCF‐7 cells was 10.44 µM, which was similar to the values obtained from derivative **5f** in the present work [16]. A series of 4‐(1,2,3‐triazol‐1‐yl)‐coumarin derivatives and investigated for their cytotoxicity against colorectal, breast, and lung cancer cells, and compounds 10a‐d showed IC_50_ scores of 9.45, >50, 36.83, and 1.72 µM, respectively, against MCF‐7 [17]. Goud and colleagues synthesized coumarin‐1,2,3‐triazole hybrids and obtained their IC_50_ values on different cancerous cells, including MDA‐MB‐231. Among the derivatives, 46 and 50 demonstrated higher cytotoxicity than the reference drug cisplatin against MDA‐MB‐231 [18]. If we compare the compound **5a,** having a methyl group 3‐position on the triazole ring, with compound **5b** containing an ethyl group at the 3‐position on the triazole nucleus, compound **5a** is found to be more active than the analog **5b** (Figure 2).

To evaluate the newly created chemicals' cytotoxicity, cisplatin, a standard chemotherapeutic, was used as the control drug. Cisplatin was selected as a reference because it is a clinically used standard chemotherapeutic and a commonly employed positive control in cytotoxicity assays on MCF‐7 and MDA‐MB‐231 cells, enabling direct benchmarking of potency and calculation of selectivity indices.

However, non‐tumorigenic cells were also cytotoxically affected by the same levels of cisplatin that caused cytotoxicity in breast cancer cells. The main objective of cancer treatment is to protect normal cells while triggering the death of cancerous cells. Derivative **5f**, which was cytotoxic to breast cancer cells, was relatively less cytotoxic to non‐tumorigenic MCF‐10A cells, indicating that they have specific cytotoxicity to breast cancer cells. To verify this specificity, the selectivity index (SI) concept has been derived from the literature [19]. An exceptional SI is defined as one with a value of 2.0 or higher. The SI of **5f** was 3.9 in MDA‐MB‐231 and 2.8 in MCF‐7 treated with derivative **5f** with a chlorine moiety. Organic chemistry recognizes the chlorine atom as a significant molecular element in the production of pharmaceuticals that are safer, more precise, and environmentally friendly [20]. Moreover, it is widely recognized that the introduction of chlorine into heterocyclic compounds enhances their pharmacological and biological effects. Further experiments were conducted using the **5f** derivative, which has an outstanding SI.

#### 2.2.2 Inhibition of Cell Cycle at S Phase via Downregulation of CDK2 and Cyclin E by Derivative **5f**

**FIGURE 3:**
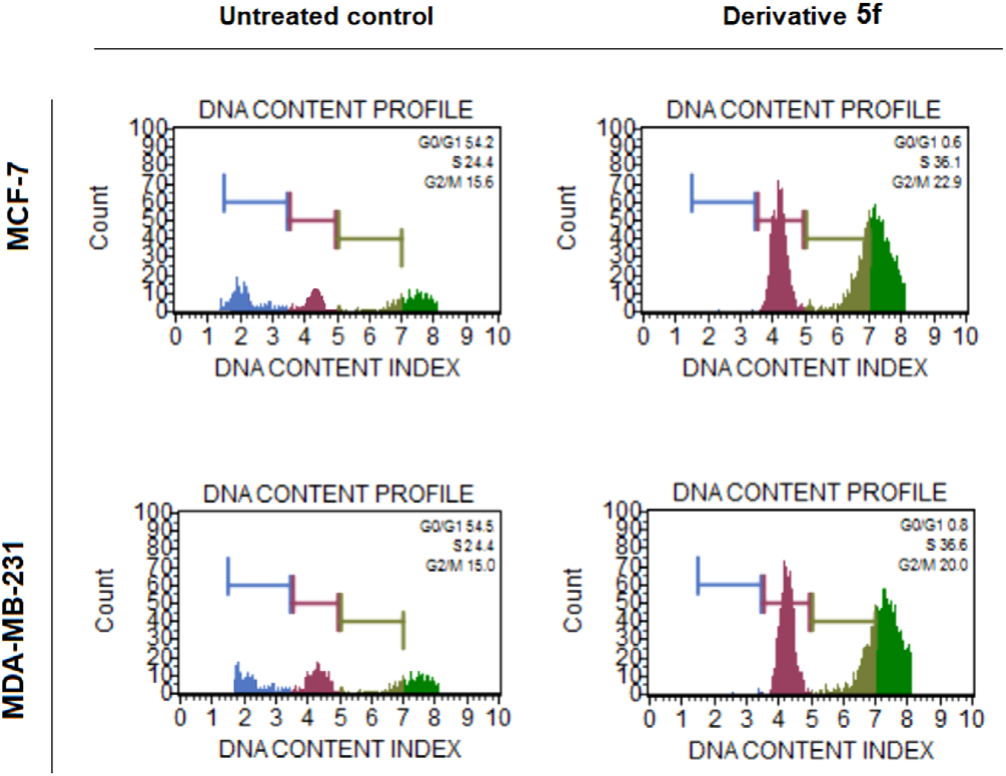
Cell cycle analysis of breast cancer cells treated with the IC_50_ value of derivative **5f** via flow cytometry. Derivative **5f** blocked the cell cycle at the S phase in both MCF‐7 and MDA‐MB‐231 breast cancer cells at 72 h.

Currently, targeted cancer treatments are aimed at blocking the basic signaling mechanisms involved in cell cycle pathways. The CDKs, which are important cell cycle pathway regulators, are rational therapeutic targets. In cancer cells, their expression is frequently disturbed, and their suppression can cause apoptosis [21]. To determine the intracellular action mechanisms of the synthesized derivatives with selective cytotoxic effects, we investigated their effects on the cell cycle. Evaluation of the cell cycle revealed that the **5f** derivative blocked both breast cancer cells in the S phase (Figure 3 and Table 2).

**TABLE 2:** Cell cycle kinetics of breast cancer cells after treatment with the IC_50_ value of derivative **5f** for 72 h. The cell block at the S phase was observed by derivative **5f**.

		Untreated Control	Derivative 5f
**MCF‐7**	**G0/G1**	54.2	0.6
	**S**	24.4	36.1
	**M**	15.6	22.9
**MDA‐MB‐231**	**G0/G1**	54.5	0.8
	**S**	24.4	36.6
	**M**	15.0	20.0

**FIGURE 4:**
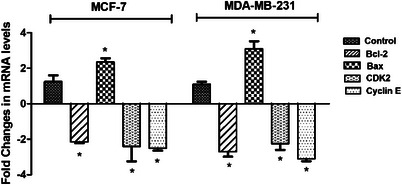
Fold changes in Bcl─2, Bax, Cyclin E, and CDK2 mRNA levels after treatment with the IC_50_ value of derivative **5f** for 72 h in MCF‐7 and MDA‐MB‐231 breast cancer cells (*p < 0.05).

Moreover, qRT‐PCR analysis showed that CDK2 and Cyclin E messenger RNA (mRNA) levels were significantly decreased by ‐2.4 and ‐2.5 fold in MCF‐7, respectively (*p* < 0.05). In MDA‐MB‐231 cells, mRNA levels of CDK2 and Cyclin E were decreased by ‐2.25 and ‐3.1 fold, respectively (*p* < 0.05) (Figure 4). By suppressing CDK2 and Cyclin E levels, the **5f** derivative successfully caused breast cancer cells to undergo cell cycle arrest at the S phase (Figure 4).

The majority of cytotoxic compounds cause DNA damage and intra‐S phase checkpoint activation, which blocks DNA synthesis and induces apoptosis or cellular senescence [22]. A number of uracil and coumarin‐based molecular hybrids were coupled with a 1,2,4‐triazole moiety and examined how they affected the cell cycle dispersion in MCF‐7 cells. Among the tested hybrids, it was found that synthesized compound 65a inhibited the cell cycle at G2/M. It was also shown that 1,2,4‐triazole‐coumarin‐glycoside hybrid induces a block in the S phase of the cell cycle [23].

#### 2.2.3 Apoptotic Induction Through Alterations in Bcl─2 and Bax mRNA Levels

Most of the new agents under development target cell apoptosis and the proteins involved in apoptosis [24]. Programmed cell death, another name for cell apoptosis, is a strictly controlled process that promotes optimal development and growth by eliminating excess immune system cells and virus‐infected or DNA‐damaged cells. Apoptotic cells produce membrane‐bound apoptotic bodies, compacted nuclear chromatin, shrinking cytoplasm, DNA fragmentation, and cleavage or degradation of many cellular proteins [25].

**FIGURE 5:**
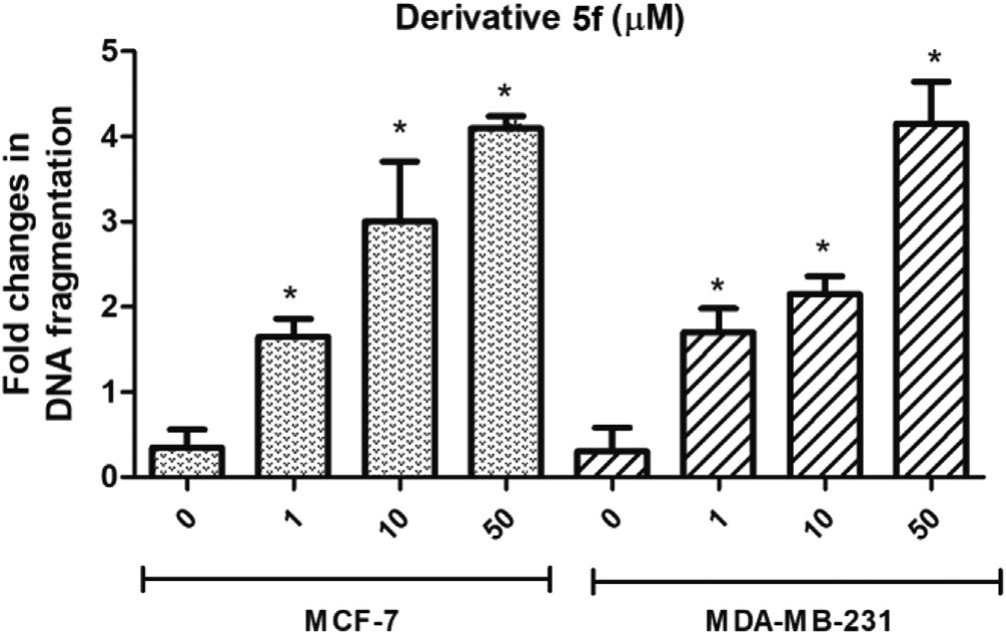
Fold changes in DNA fragmentation in breast cancer cells after treatment with derivative **5f** for 72 h (*p < 0.05).

To determine whether the **5f** derivative, which was found to have a selective cytotoxicity, induces apoptosis in breast cancerous cells, the amount of DNA fragmentation inside tumor cells was measured. As shown in Figures 5, 5**f** derivative induced concentration‐dependent DNA fragmentation in both MDA‐MB‐231 and MCF‐7 at 72 h. Pretreatment of MCF‐7 with 1, 10, and 50 µM derivative **5f** induced DNA fragmentation by 1.65‐, 3‐, and 4.1‐fold, respectively (*p* < 0.05). For MDA‐MB‐231, DNA fragmentation was induced by 1.7‐, 2.15‐, and 4.15‐fold after treatment with 1, 10, and 50 µM derivative **5f**, respectively (*p* < 0.05).

To ensure that apoptotic cell death has been induced, mRNA expression levels of Bcl─2 family proteins Bcl─2 and Bax were investigated via the quantitative reverse transcription polymerase chain reaction (qRT‐PCR) method. Bcl─2 family proteins are the basic players that regulate the mitochondrial apoptotic signal pathway [26]. The Bcl─2 family has 3 different functional protein groups, namely: i) anti‐apoptotic proteins (Bcl─2, Bcl─XL, Bcl─W, Mcl‐1, and BFL‐1/A1), ii) pro‐apoptotic pore‐formers (BAX, BAK, and BOK), and iii) pro‐apoptotic BH3‐only proteins (BAD, BID, BIK, BIM, BMF, HRK, NOXA, and PUMA) [27].

It was shown that the **5f** derivative down‐regulated the mRNA amounts of Bcl─2 and up‐regulated the mRNA levels of Bax in both breast cancer cells (Figure 4). For MCF‐7, levels of Bcl─2 were downregulated by 2.1‐fold, and Bax levels were upregulated by 2.35‐fold after treatment with derivative **5f** (Figure 4). For MDA‐MB‐231, levels of Bcl─2 were downregulated by 3.1‐fold, and Bax levels were upregulated by 2.35‐fold (Figure 4). El‐Sayed et al. screened the mitochondria‐mediated apoptosis of novel 1,2,4‐triazole‐coumarin‐glycoside hybrids and showed that derivatives trigger apoptosis in MCF‐7 and upregulate Bax while inhibiting Bcl─2 [23]. Banerji et al. employed several triazole‐substituted quinazoline hybrids and screened their apoptotic effects on different cancer cells. The findings indicated that Compound **5b** altered the mitochondrial membrane potential in MCF‐7, causing apoptotic cell death [28].

#### 2.2.4 Docking With CDK2 and Bcl─2 Proteins

To elucidate the mechanism of anticancer activity, we employed the docking protocol for all the synthesized compounds and analyzed the docking scores and interaction modes at the target protein binding sites. The interaction residues and docking outcomes of the given compounds against the Bcl─2 and CDK2/Cyclin‐E complex proteins are displayed in Table 3. Compound **5f** emerged as a promising inhibitor of CDK2 and Bcl─2 within the novel triazole‐coumarin hybrid derivatives because of its positive associations and docking scores in protein binding sites.

**TABLE 3:** Docking scores (in kcal/mol) of novel triazole‐coumarin hybrid derivatives and interacting residues at the active site of CDK2 and Bcl─2.

	Title	Docking scores	Hydrogen bonds/distance (Å)	Hydrophobic interactions
***Bcl─2***	5a	−6.71	GLN118 (2.12)	PHE104, PHE112, LEU137, ALA149, GLU152, VAL156, ARG110 (pi‐Cation)
	5b	−6.58	GLN118 (1.99)	PHE104, ASP11, MET115, ALA149, PHE153, VAL156
	5c	−8.16	ASP140 (1.95)	PHE104, ASP11, GLU136, ALA149, TYR108, ARG146
	5d	−8.29	ASP140 (1.97)	PHE104, ASP11, GLU136, LEU137, ALA149, ARG146, TYR108 (pi‐Stacking)
	5e	−7.23	ASN143 (1.80)	PHE104, TYR108, VAL133, LEU137, ARG146 (Salt Bridges)
	5f	−9.04	ARG146 (1.70)	MET115, VAL133, GLU136, LEU137, ALA149, ASP140, TYR108 (pi‐Stacking)
***CDK2/Cyclin‐E***	5a	−10.54	LEU83 (2.37), LYS89 (2.51), LYS20 (2.32)	PHE82 (pi‐Stacking), ALA31, ILE10, PHE80, LEU134, HIS84, GLN85
	5b	−9.73	LEU83 (1.93), LEU83 (2.52), ASP86 (2.26)	PHE80, ALA31, ILE10, PHE82, VAL18, ASP86, HIS84, GLN85
	5c	−9.35	LEU83 (2.84), LYS89 (1.91), ASP86 (1.86)	PHE82 (pi‐Stacking), ALA31, ILE10, PHE80, LEU134, HIS84, GLN85
	5d	−8.60	LEU83 (1.88), LEU83 (2.44), ASP86 (2.16)	LYS20 (pi‐Stacking), LYS89 (pi‐Stacking), ILE10, ALA31, PHE82, ALA144, PHE80
	5e	−8.54	GLU12 (2.13), LYS33 (2.52)	PHE80 (pi‐Stacking), ALA31, ILE10, PHE82, LEU83, ASP86, HIS84, GLN85
	5f	−11.16	LEU83 (1.81), LEU83 (2.32), ASP86 (2.46)	ALA31, PHE82, ILE10, LYS89 (pi‐Stacking), LYS20 (pi‐Stacking)

**FIGURE 6:**
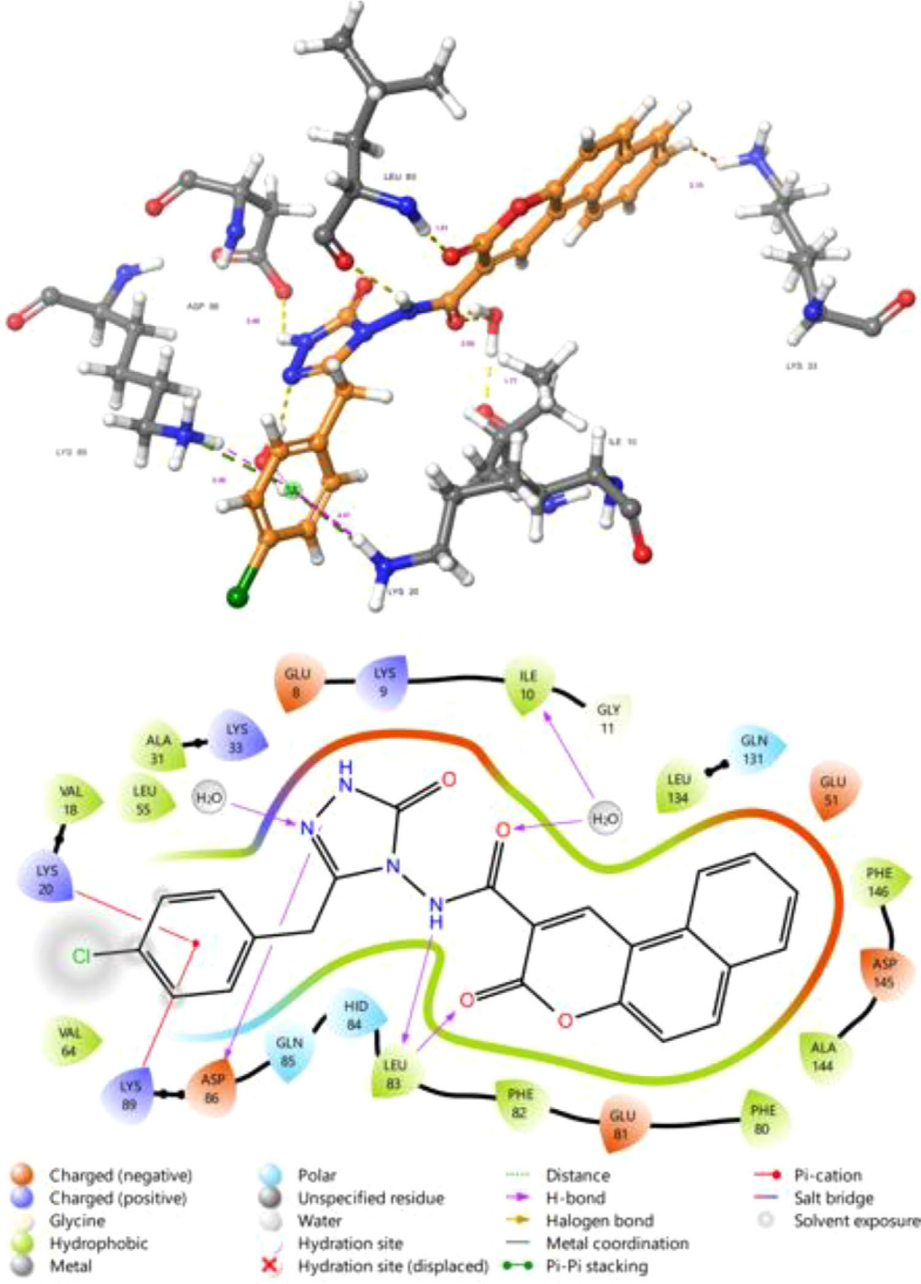
2D and 3D interaction diagrams of compound **5f** at the active site of CDK2/Cyclin E complex.

As a potential CDK2 inhibitor, Figure 6 shows a detailed insight into the molecular interactions occurring between the CDK2 protein (PDB code 7KJS) and compound 5f. This analysis is crucial for understanding the potential inhibitory mechanism of the compound on the CDK2 enzyme and for developing strategies aimed at the rational design of more effective inhibitors. Compound **5e** binds to the active site of CDK2 through a variety of non‐covalent interactions. Hydrogen bonds play a pivotal role in the binding stability of the compound. The carbonyl oxygen atom of the coumarone ring of the compound forms a hydrogen bond with the backbone of the LEU83 residue. Additionally, the ‐NH‐ group of the compound **5f** also establishes a hydrogen bond interaction with the carbonyl oxygen of LEU83. One of the pyrazolone nitrogen atoms of the compound forms a hydrogen bond with ASP86. This intricate network of hydrogen bonds ensures optimal positioning of the ligand within the active site and enhances its binding affinity. Hydrophobic interactions are a significant factor in the accommodation of compound **5f** within the active site pocket. The chlorophenyl ring of the compound exhibits extensive hydrophobic interactions with hydrophobic amino acid residues such as VAL 18, LYS 20, VAL 64, and LYS 89. The coumarone ring, on the other hand, is surrounded by hydrophobic and partially polar residues, including ASP 145, ALA 144, PHE 80, GLU 81, PHE 82, and LEU 83. These widespread hydrophobic interactions contribute to the tight fit of the compound within the active site and optimize its binding affinity. T‐shaped pi‐stacking interaction is noted between the chlorophenyl ring of the compound and LYS89 and LYS20, signifying specific interactions that can further contribute to binding stability. Notably, the majority of these hydrophobic interactions involved leucine amino acids, which are pivotal hydrophobic residues.

Compound **5e** ranked as the second‐most effective among the series due to its high binding affinity and connections with the CDK2 active site. Compound **5e** formed two hydrogen bonds with the GLU12 and LYS33 amino acid residues, with bond distances measuring 2.13 and 2.52 Å, respectively. The pi‐stacking interaction was also observed between compound **5e** and the PHE80 residue. Additionally, hydrophobic interactions with the CDK2 protein were evident, involving residues such as ALA31, ILE10, PHE82, LEU83, ASP86, HIS84, and GLN85.

**FIGURE 7:**
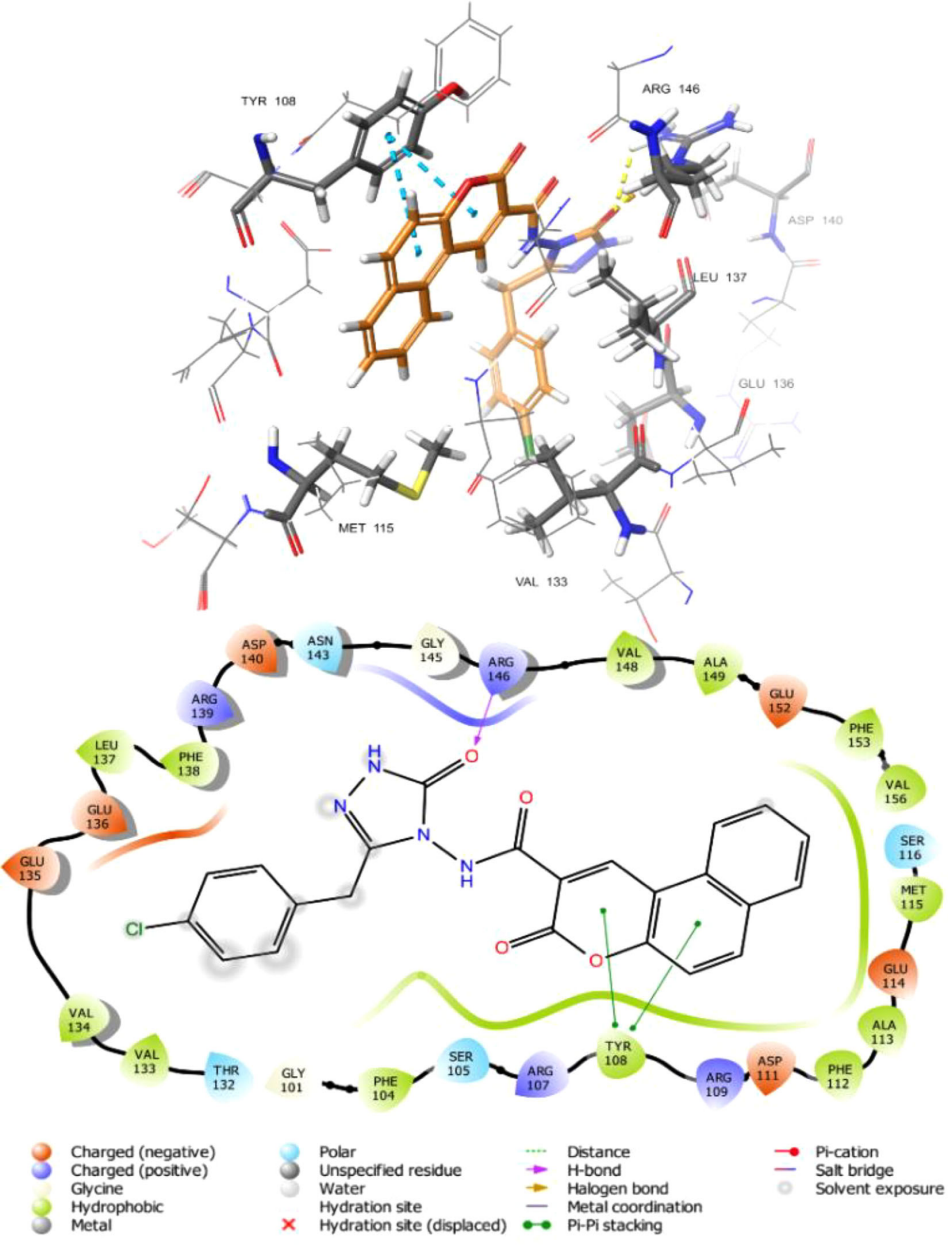
2D and 3D interaction diagrams of compound **5f** at the active site of Bcl─2.

As an intriguing inhibitor of Bcl─2, compound **5f** formed a strong hydrogen bond with the ARG146 with bond lengths of 1.70 Å and a pi‐pi stacking interaction between the TYR108 amino acid residue and the coumarin rings. Additionally, hydrophobic bonds within compound **5f** and the Bcl─2 protein's binding site residues were noted, including MET115, VAL133, GLU136, LEU137, ALA149, and ASP140. Figure 7 illustrates compound **5f**'s 2‐D and 3‐D interactions with the Bcl─2 protein's active site residues.

Compound 5f binds to the active site of the CDK2 and Bcl─2 protein through a combination of strong hydrogen bonds, extensive hydrophobic interactions, and pi‐stacking interactions. This multi‐modal interaction profile supports the potential of the compound to inhibit the CDK2/Cyclin E complex and Bcl─2. The detailed binding profile presented herein provides fundamental structural insights for the rational design of novel and more effective anticancer drugs targeting CDKs and Bcl─2.

## 3 Conclusion

We have developed a new protocol for synthesizing triazole‐coumarin hybrids (**5a–f**) and successfully characterized them. The synthesized compounds were evaluated for their anticancer potential against MDA‐MB‐231 and MCF‐7 human breast cancer cell lines and non‐tumorigenic MCF‐10A human breast cells. Among them, derivative **5f** exhibited the highest cytotoxic activity against breast cancer cells while demonstrating lower toxicity toward non‐tumorigenic cells. Mechanistically, derivative **5f** exerted its cytotoxic effect by inducing apoptosis and arresting the cell cycle at the S phase, accompanied by the downregulation of CDK2 and Cyclin E and modulation of Bcl─2 and Bax expression. Additionally, molecular docking studies indicated favorable interactions of **5f** with CDK2 and Bcl─2 proteins, supporting its potential role in breast cancer treatment. Overall, this study highlights the potential of triazole‐coumarin hybrids as promising candidates for anticancer drug development, with derivative **5f** warranting further investigation.

## 4 Experimental

### 4.1 Chemistry

Alfa Aesar and Merck provided the chemicals. A Stuart SMP30 melting point device was utilized to measure the uncorrected melting points in capillary tubes. Using dimethyl sulfoxide (DMSO)‐d*_6_* as the solvent, ^1^H and ^13^C nuclear magnetic resonance (NMR) spectra were captured using a Varian‐Mercury 400 spectrometer (Supporting Information). Using the Thermo Scientific Quantum Access Max liquid chromatography‐mass spectrometry (LC‐MS) spectrometer, the mass spectra of the produced compounds were acquired. Elemental compositions were analyzed using a Carlo Erba 1106 CHN analyzer, with experimental results matching calculated values within ±0.4%. The related literature was followed in the synthesis of starting compounds **1a–f** and **3** [14, 29].

### 4.2 Synthesis of Compounds **5a–f**

Compound **4** (0.01 mol, 1 equiv.) was added to a solution of compound **2a** (or **2b‐f**, 0.01 mol, 1 equiv) in benzene (10 mL). The mixture was refluxed for 2 h. The reaction progress was monitored by TLC (ethyl acetate:hexane, 2:1, silica gel). After cooling to room temperature, a white solid product formed, which was filtered and washed with ethanol.

#### 4.2.1 *N*‐(3‐Methyl‐5‐oxo‐1,5‐dihydro‐4*H*‐1,2,4‐triazol‐4‐yl)‐3‐oxo‐3*H*‐benzo[*f*]chromene‐2‐carboxamide (**5a**)

Yield: 2.69 g (80 %), melting point (m.p.) 335–336°C, IR (*v*_max_/cm^−1^): 3236 (NH), 1720, 1693, 1675 (C ═ O), 1568 (C ═ N), 1204 (C─O). ^1^H‐NMR (400 MHz, DMSO‐*d_6_*), δ, ppm: 2.08 (3H, s, CH_3_); 7.55 (1H, d, *J* = 8.0 Hz, Ar─H); 7.63 (1H, t, *J* = 8.0 Hz, Ar─H); 7,72 (1H, t, *J* = 8,0 Hz, Ar─H); 8.01 (1H, d, *J* = 8 Hz, Ar─H); 8.26 (1H, d, *J* = 8 Hz, Ar─H); 8.43 (1H, d, *J* = 8 Hz, Ar─H); 9.31 (1H, s, CH, coumarin‐H4); 10.95 (1H, s, CONH); 11.59 (1H, s, NH). ^13^C APT (100 MHz, DMSO‐*d_6_*), δ, ppm: 11.11 (CH_3_); 112.75, 116.16, 116.75, 123.61, 127.16, 129.26, 129.49, 129.66, 130.27, 136.97 (Ar─C); 144.85 (CH, coumarin‐C4); 145.46 (C ═ N); 152.92 (coumarin‐C3); 155.27 (C ═ O); 159.51 (C ═ O); 161.94 (C ═ O). LC‐MS, *m*/*z*: 336.98 [M + H]^+^. Elemental analysis for C_17_H_12_N_4_O_4_: C, 60.71; H, 3.60; N, 16.66; Found: C, 60.87; H, 3.65; N, 16.73.

#### 4.2.2 *N*‐(3‐Ethyl‐5‐oxo‐1,5‐dihydro‐4*H*‐1,2,4‐triazol‐4‐yl)‐3‐oxo‐3*H*‐benzo[*f*]chromene‐2‐carboxamide (**5b**)

Yield: 2.8 g (80 %), m.p.: 314–315°C, IR (*v*_max_/cm^−1^): 3250, 3183 (NH), 1718, 1671 (C ═ O), 1596 (C ═ N), 1206 (C─O). ^1^H‐NMR (400 MHz, DMSO‐*d_6_*), δ, ppm: 1.13 (3H, t, *J =* 4 Hz, CH_3_); 2.42 (2H, q, *J =* 4 Hz, CH_2_), 7.59 (1H, d, *J* = 8.0 Hz, Ar─H); 7.64 (1H, t, *J* = 8.0 Hz, Ar─H); 7.74 (1H, t, *J* = 8,0 Hz, Ar─H); 8.05 (1H, d, *J* = 8 Hz, Ar─H); 8.29 (1H, d, *J* = 8 Hz, Ar─H); 8.49 (1H, d, *J* = 8 Hz, Ar─H); 8.36 (1H, s, CH, coumarin‐H4); 10.96 (1H, s, CONH); 11.60 (1H, s, NH). ^13^C APT (100 MHz, DMSO‐*d_6_*), δ, ppm:10.11 (CH_3_); 18.46 (CH_2_); 112.82, 116.36, 116.80, 122.70, 127.15, 128.74, 129.50, 129.67, 129.93, 130.32, 136.98 (Ar─C); 144.89 (CH, coumarin‐C4); 149.23 (C ═ N); 153.07 (coumarin‐C3); 155.32 (C ═ O); 159.49 (C ═ O); 162.00 (C ═ O). LC‐MS, *m*/*z*: 351.03 [M + H]^+^. Elemental analysis for C_18_H_14_N_4_O_4_: C, 61.71; H, 4.03; N, 15.99; Found: C, 61.85; H, 4.08; N, 16.07.

#### 4.2.3 3‐Oxo‐*N*‐(5‐oxo‐3‐phenyl‐1,5‐dihydro‐4*H*‐1,2,4‐triazol‐4‐yl)‐3*H*‐benzo[*f*]chromene‐2‐carboxamide (**5c**)

Yield: 3.38 (85 %), m.p.: 320–321°C, IR (*v*_max_/cm^−1^): 3269, 3180 (NH), 1711, 1684 (C ═ O), 1568 (C ═ N), 1205 (C─O). ^1^H‐NMR (400 MHz, DMSO‐*d_6_*), δ, ppm: 7.45–7.48 (4H, m, Ar─H); 7.54 (1H, t, *J* = 8.0 Hz, Ar─H); 7.64 (1H, t, *J* = 8,0 Hz, Ar─H); 7.80–7.83 (2H, m, Ar─H); 7.94 (1H, d, *J* = 8 Hz, Ar─H); 8.19 (1H, d, *J* = 12 Hz, Ar─H); 8.32 (1H, d, *J* = 8 Hz, Ar─H); 9.19 (1H, s, CH, coumarin‐H4); 11.26 (1H, s, CONH); 12.18 (1H, s, NH). ^13^C APT (100 MHz, DMSO‐*d_6_*), δ, ppm:112.70, 115.70, 116.68, 122.52, 126.64, 127.11, 127.38, 127.84, 128.73, 129.18, 129.21, 129.40, 129.61, 130.17, 130.75, 137.02 (Ar─C); 145.20 (CH, coumarin‐C4); 146.27 (C ═ N); 153.22 (coumarin‐C3); 155.29 (C ═ O); 159.51 (C ═ O); 161.88 (C ═ O). LC‐MS, *m*/*z*: 399.07 [M + H]^+^. Elemental analysis for C_22_H_14_N_4_O_4_: C, 66.33; H, 3.54; N, 14.06; Found: C, 66.41; H, 3.60; N, 14.15.

#### 4.2.4 *N*‐(3‐Benzyl‐5‐oxo‐1,5‐dihydro‐4*H*‐1,2,4‐triazol‐4‐yl)‐3‐oxo‐3*H*‐benzo[*f*]chromene‐2‐carboxamide (**5d**)

Yield: 3.46 g (84 %), m.p.: 258–259°C, IR (*v*_max_/cm^−1^): 3247, 3181 (NH), 1746, 1711, 1683 (C ═ O), 1563 (C ═ N), 1212 (C─O). ^1^H‐NMR (400 MHz, DMSO‐*d_6_*), δ, ppm: 4.01 (2H, s, CH_2_); 7.17 (1H, d, *J* = 8.0 Hz, Ar─H); 7.22–7.28 (5H, m, Ar─H); 7.55 (1H, d, *J* = 8.0 Hz, Ar─H); 8.06 (1H, d, *J* = 8,0 Hz, Ar─H); 8.10 (1H, d, *J* = 8 Hz, Ar─H); 8.27 (1H, d, *J* = 8 Hz, Ar─H); 8.36 (1H, d, *J* = 8 Hz, Ar─H); 8.82 (1H, s, CH, coumarin‐H4); 10.92 (1H, s, CONH); 11.76 (1H, s, NH). ^13^C APT (100 MHz, DMSO‐*d_6_*), δ, ppm: 30.15 (CH_2_); 112.78, 116.90, 117.70, 122.74, 127.03, 127.68, 128.76, 129.11, 129.48, 129.62, 130.25, 134.57, 136.18 (Ar─C); 144.40 (CH, coumarin‐C4); 153.59 (C ═ N); 155.07 (coumarin‐C3); 157.21 (C ═ O); 159.21 (C ═ O); 161.69 (C ═ O). LC‐MS, *m*/*z*: 413.07 [M + H]^+^. Elemental analysis for C_23_H_16_N_4_O_4_: C, 66.99; H, 3.91; N, 13.59; Found: C, 67.13; H, 3.98; N, 14.05.

#### 4.2.5 *N*‐(3‐(4‐Methylbenzyl)‐5‐oxo‐1,5‐dihydro‐4*H*‐1,2,4‐triazol‐4‐yl)‐3‐oxo‐3*H*‐benzo[*f*]chromene‐2‐carboxamide (**5e**)

Yield : 3.53 g (83 %), m.p.: 238–239°C, IR (*v*_max_/cm^−1^): 3251, 3178 (NH), 1734, 1718, 1673 (C ═ O), 1569 (C ═ N), 1214 (C─O). ^1^H‐NMR (400 MHz, DMSO‐*d_6_*), δ, ppm: 2.16 (3H, s, CH_3_); 3.76 (2H, s, CH_2_); 7.03 (2H, d, *J* = 8.0 Hz, Ar─H); 7.13 (2H, d, *J* = 8.0 Hz, Ar─H); 7.59 (1H, d, *J* = 8,0 Hz, Ar─H); 7.64 (1H, t, *J* = 8 Hz, Ar─H); 7.74 (1H, t, *J* = 8 Hz, Ar─H); 8.04 (1H, d, *J* = 8 Hz, Ar─H); 8.29 (1H, d, *J* = 8 Hz, Ar─H); 8.43 (1H, t, *J* = 8 Hz, Ar─H); 9.23 (1H, s, CH, coumarin‐H4); 10.95 (1H, s, CONH); 11.71 (1H, s, NH). ^13^C APT (100 MHz, DMSO‐*d_6_*), δ, ppm:21.03 (CH_3_), 30.87 (CH_2_); 112.74, 116.39, 116.800, 122.59, 127.16, 129.29, 129.33, 129.39, 129.53, 129.67, 130.19, 130.32, 132.16, 136.94 (Ar─C); 144.57 (CH, coumarin‐C4); 147.43 (C ═ N); 152.94 (coumarin‐C3); 155.26 (C ═ O); 159.40 (C ═ O); 161.79 (C ═ O). LC‐MS, *m*/*z*: 427.08 [M + H]^+^. Elemental analysis for C_24_H_18_N_4_O_4_: C, 67.60; H, 4.25; N, 13.14; Found: C, 67.71; H, 4.29; N, 13.19.

#### 4.2.6 *N*‐(3‐(4‐Chlorobenzyl)‐5‐oxo‐1,5‐dihydro‐4*H*‐1,2,4‐triazol‐4‐yl)‐3‐oxo‐3*H*‐benzo[*f*]chromene‐2‐carboxamide (**5f**)

Yield: 3.79 g (85 %), m.p.: 335–336°C, IR (*v*_max_/cm^−1^): 3247, 3180 (NH), 1717, 1673 (C ═ O), 1567 (C ═ N), 1214 (C─O), 744 (C─Cl). ^1^H‐NMR (400 MHz, DMSO‐*d_6_*), δ, ppm: 3.82 (2H, s, CH_2_); 7.29‐7.33 (4H, m, Ar─H);7.62 (2H, t, *J* = 8.0 Hz, Ar─H); 7.76 (1H, d, *J* = 8,0 Hz, Ar─H); 8.06 (1H, d, *J* = 8 Hz, Ar─H); 8.29 (1H, d, *J* = 8 Hz, Ar─H); 8.47 (1H, d, *J* = 8 Hz, Ar─H); 9.27 (1H, s, CH, coumarin‐H4); 10.97 (1H, s, CONH); 11.75 (1H, s, NH). ^13^C APT (100 MHz, DMSO‐*d_6_*), δ, ppm: 30.55 (CH_2_); 112.79, 116.36, 116.82, 122.64, 127.18, 128.71, 129.33, 129.54, 129.69, 130.34, 131.42, 131.92, 134.30, 137.00 (Ar─C); 144.69 (CH, coumarin‐C4); 147.03 (C ═ N); 152.88 (coumarin‐C3); 155.32 (C ═ O); 159.53 (C ═ O); 161.81 (C ═ O). LC‐MS, *m*/*z*: 446.97 [M(Cl^35^)+ H]^+^, 448.98 [M(Cl^37^)+ H]^+^. Elemental analysis for C_23_H_15_ClN_4_O_4_: C, 61.82; H, 3.38; N, 12.54; Found: C, 61.95; H, 3.43; N, 12.59.

### 4.3 Cell Culture

Human cell lines, breast cancer MCF‐7 and MDA‐MB‐231, along with the human breast epithelial cell line (MCF‐10A), were purchased from ATCC (Rockville, USA). The necessary supplies, including fetal bovine serum (FBS), RPMI 1640 medium, 3‐(4,5‐dimethylthiazol‐2‐yl)‐2,5‐diphenyltetrazolium bromide (MTT), and DMSO, were provided by Merck (Darmstadt, Germany). The cells were maintained at 37°C in a CO_2_─enriched chamber, utilizing RPMI 1640 medium fortified with 10% FBS and 100 U/mL of penicillin/streptomycin [30].

### 4.4 Cell Viability Assessment

Cell survival was evaluated using the MTT technique, which measures cellular redox activity. In metabolically active cells, MTT (3‐(4,5‐dimethylthiazol‐2‐yl)) is reduced to form an insoluble purple formazan. This formazan was solubilized, and its concentration was quantified by measuring optical density. MTT, sourced as a powder from Sigma, was made with phosphate‐buffered saline (PBS) in a 5 mM standard solution. The density at which cells were propagated was 10⁴ cells per well in 96‐well plates. Triazole‐coumarin hybrids were dissolved in DMSO, and varying concentrations (0.1–200 µM) of these derivatives were applied to the cells. Following treatment for 24, 48, and 72 h, 20 µL of the MTT mix was applied to each well, and the wells were then incubated for 4 h at thirty‐seven degrees in a CO_2_ incubator. After incubating, the formazan crystals were dissolved by adding 200 µL of DMSO after the MTT mixture was withdrawn. The absorption rate was recorded at 590 nm utilizing a Tecan spectrophotometer [31]. Cisplatin served as the reference drug. The concentration needed to 50% decrease cell viability is demonstrated by the IC_50_ values, which were estimated employing CalcuSyn 2.0 software (Biosoft). To determine the selective toxicity of the triazole‐coumarin hybrids against breast cancer cells, the formula was used to compute the SI = IC_50_ for normal cells/IC_50_ for cancer cells [32].

### 4.5 DNA Fragmentation Analysis

As an apoptotic signature, DNA fragmentation was evaluated using the Cell Death Detection kit (Sigma). In 96‐well plates, cancerous cells were cultivated at a density of 1×10⁵ cells per well, and compound **5f** was applied at its IC_50_ concentration for 72 h. In brief, 20 µL of cell lysates were transferred to streptavidin‐coated wells preloaded with anti‐DNA antibodies and held at RT for 2 h. Three washes afterward with the provided washing buffer, 2,2‐azino‐di‐(3‐ethylbenzthiazoline sulfonate) diammonium salt was put into every well. Absorbance was determined at 405 nm employing a spectrophotometer [33].

### 4.6 PCR Reaction and Gene Expression Analysis

To figure out the mRNA levels of apoptosis‐related proteins B‐cell lymphoma 2 (Bcl─2), Bcl─2‐associated X protein (Bax), cyclin‐dependent kinase 2 (CDK2), and Cyclin E, total mRNA was extracted from cells treated with compound **5f** using Trizol reagent (Sigma). Complementary DNA (cDNA) synthesis was conducted from total mRNA using a Qiagen cDNA synthesis kit as described by the manufacturer. The primers listed below (350 nM concentration) were utilized: Bcl─2; forward (GGTGCCACCTGTGGTCCACCTG); reverse (CTTCACTTGTGGCCCAGATAG), Bax; forward (ATGGACGGGTCCGGGGAGCAGC); reverse (CCCCAGTTGAAGTTGCCGTCAG), CDK2; forward (AGTACACCTGCTGTCCTTCT); reverse (TGGCTGAAATCCGCTTGTT), Cyclin E; forward (CTCCAGGAAGAGGAAGGCAA); reverse (TCGATTTTGGCCATTTCTTCA), human GAPDH; forward (GGCAAATTCAACGGCACAGT); reverse (AGATGGTGATGGGCTTCCC). The PCR reaction mixture consisted of 1X buffer, 0.2 mM dNTPs, 1.6 mM MgCl_2_, 50 pmol of primer sets, cDNA, and 0.25 U of Taq polymerase. The thermal cycling conditions included an initial denaturation at 95°C for 60 s, followed by 50 cycles comprising 15 s of denaturation at 95°C, 15 s of annealing at 60°C, and 60 s of extension at 72°C. The median CT value of GAPDH, the naturally present housekeeping gene, was utilized to normalize the cycle threshold (CT) values. Levels of compared transcription were established using the comparative ^ΔΔ^CT method [30].

### 4.7 Cell Cycle Analysis

In 24‐well plates, cells were introduced at a concentration of 5×10⁵ colonies per plate, and compound **5f** was administered at its IC_50_ concentration for 72 h. The cell cycle phases were evaluated using the Cayman Cell Cycle Phase Determination Kit. Following treatment, applying cold PBS, cells were rinsed, trypsinized, and 5 min of centrifugation at 500 × *g*. The resulting pellets were resuspended in 250 µL of fixative and held at −20°C for 2 h. After a second centrifugation at 500 × *g* for 5 min, washing was carried out with PBS and stained with propidium iodide (PI) for 30 min at room temperature. Flow cytometric analysis of the cell cycle was implemented using a BD Accuri C6 flow cytometer, with detection wavelengths ranging from 488 to 650 nm [31].

### 4.8 Molecular Docking and Interaction Analysis

The crystal structures of the ligand‐binding domain of the CDK2/Cyclin‐E complex (PDB code: 7KJS) and BCL‐2 protein (PDB code: 6O0K) were obtained from the Protein Data Bank at a resolution of 2.19 Å and 1.62 Å, respectively [34, 35]. The Protein Preparation Wizard was executed to prepare the proteins within the Schrodinger Maestro software suite [36]. Missing side chains were subsequently added, and water molecules were eliminated from the crystallographic structure, except for those within 5 Å of the binding site. Energy minimization and protein refinement were performed with a root‐mean‐square deviation (RMSD) tolerance of up to 0.3 Å using the OPLS‐2005 force field, with residues protonated at physiological pH.

The chemical configurations of all newly synthesized compounds were drawn and tuned using Gaussian 09 W [37]. The optimized structures were stored in SDF format and imported into the Maestro graphical user interface (GUI). Each compound was prepared using the default settings in the LigPrep module, applying the OPLS 2005 force field at physiological pH. Subsequently, every configuration was docked into the receptor using a 20 Å grid.

Docking simulations were carried out utilizing the induced fit docking approach, integrated with the Glide/XP technique, all within the Schrodinger Maestro molecular modeling suite. The co‐crystallized ligands were utilized to define the binding site of the target and subsequently redocked into this site [36, 37]. Root mean square deviation values of less than 2 Å confirmed the accuracy and reliability of the docking procedure [38]. Non‐covalent interactions between the ligand and the CDK2 protein were analyzed using the Protein‐Ligand Interaction Profiler online tool [39].

### 4.9 Statistical Analysis

Statistical evaluations were performed using GraphPad Prism 5.0. To determine notable variations between the groups being tested, a one‐way analysis of variance (ANOVA) was employed. This method assesses variability within and between groups to identify whether observed differences are statistically meaningful. Following the ANOVA, Tukey's post‐hoc test was implemented for pairwise multiple comparisons to pinpoint specific group differences while controlling for type I error. A *p*‐value threshold of less than 0.05 was regarded as an indicator of statistical significance. The data were presented as the mean ± standard deviation, ensuring clarity and reliability in the representation of experimental variability.

## Conflicts of Interest

The authors declare no conflicts of interest.

## Supporting information




**Supporting File 1**: cbdv70424‐sup‐0001‐SuppMat.doc

## Data Availability

The data that support the findings of this study are available on request from the corresponding author. The data are not publicly available due to privacy or ethical restrictions.
