# Supplementary material for: Design, Synthesis, and Molecular Docking of Triazole‐Coumarin Hybrids as Potent Breast Cancer Inhibitors Targeting Cell Cycle and Apoptosis
Source: Chem Biodivers. 2025 Sep 5;22(12):e01775. doi: 10.1002/cbdv.202501775 (PMC12716005; doi:10.1002/cbdv.202501775)
Supplement: Supplementary file 1 — Supporting File 1: cbdv70424‐sup‐0001‐SuppMat.doc [file CBDV-22-e01775-s001.doc]

| Comp. No. | **InChI** |
| --- | --- |
| **5a** | InChI=1S/C17H12N4O4/c1-9-18-19-17(24)21(9)20-15(22)13-8-12-11-5-3-2-4-10(11)6-7-14(12)25-16(13)23/h2-8H,1H3,(H,19,24)(H,20,22) |
| **5b** | InChI=1S/C18H14N4O4/c1-2-15-19-20-18(25)22(15)21-16(23)13-9-12-11-6-4-3-5-10(11)7-8-14(12)26-17(13)24/h3-9H,2H2,1H3,(H,20,25)(H,21,23) |
| **5c** | InChI=1S/C22H14N4O4/c27-20(25-26-19(23-24-22(26)29)14-7-2-1-3-8-14)17-12-16-15-9-5-4-6-13(15)10-11-18(16)30-21(17)28/h1-12H,(H,24,29)(H,25,27) |
| **5d** | InChI=1S/C23H16N4O4/c28-21(26-27-20(24-25-23(27)30)12-14-6-2-1-3-7-14)18-13-17-16-9-5-4-8-15(16)10-11-19(17)31-22(18)29/h1-11,13H,12H2,(H,25,30)(H,26,28) |
| **5e** | InChI=1S/C24H18N4O4/c1-14-6-8-15(9-7-14)12-21-25-26-24(31)28(21)27-22(29)19-13-18-17-5-3-2-4-16(17)10-11-20(18)32-23(19)30/h2-11,13H,12H2,1H3,(H,26,31)(H,27,29) |
| **5f** | InChI=1S/C23H15ClN4O4/c24-15-8-5-13(6-9-15)11-20-25-26-23(31)28(20)27-21(29)18-12-17-16-4-2-1-3-14(16)7-10-19(17)32-22(18)30/h1-10,12H,11H2,(H,26,31)(H,27,29) |


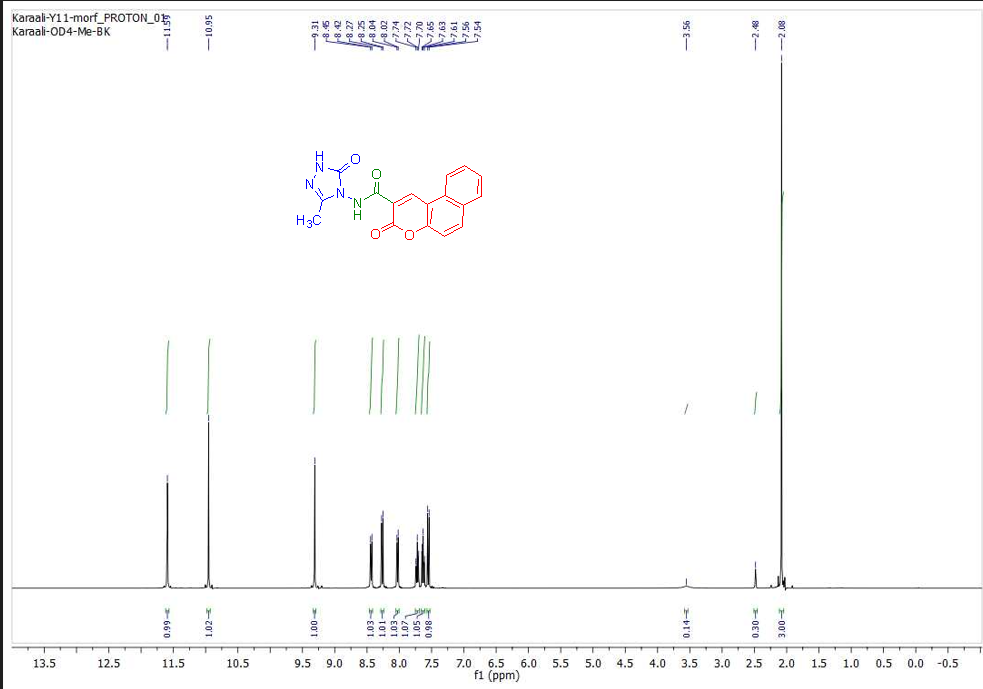


^1^H-NMR spectra of compound **5a**


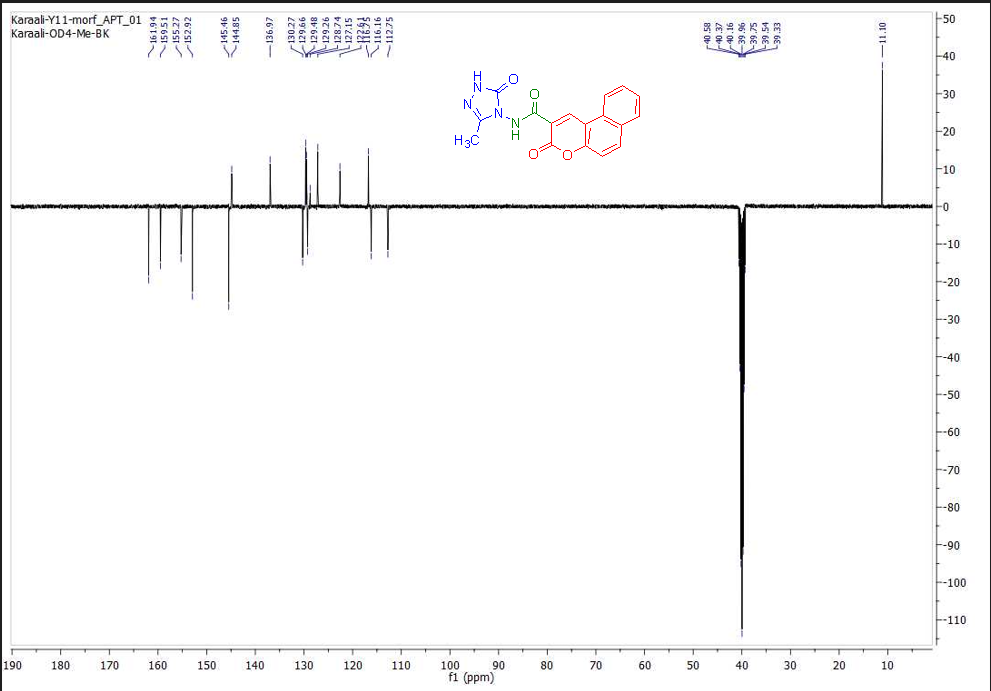


^13^C-NMR spectra of compound **5a**

**
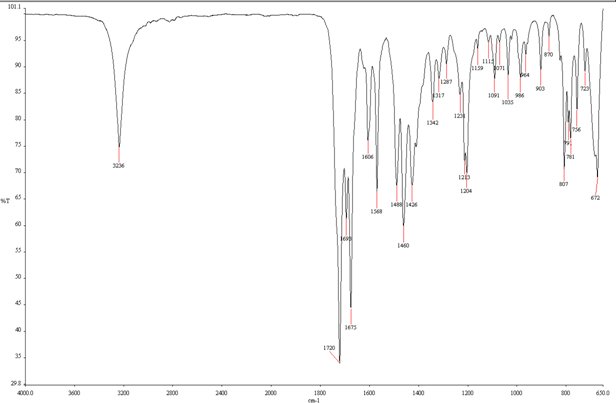
**

IR spectra of compound **5a**

**
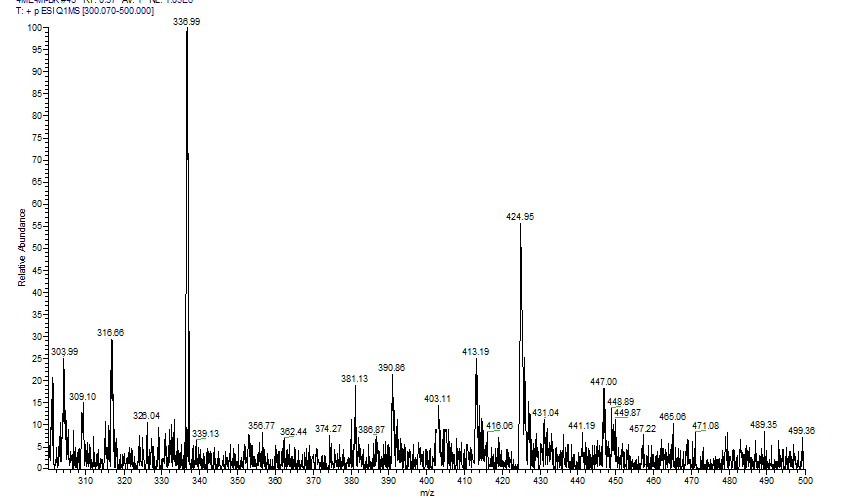
**

MS spectra of compound **5a**


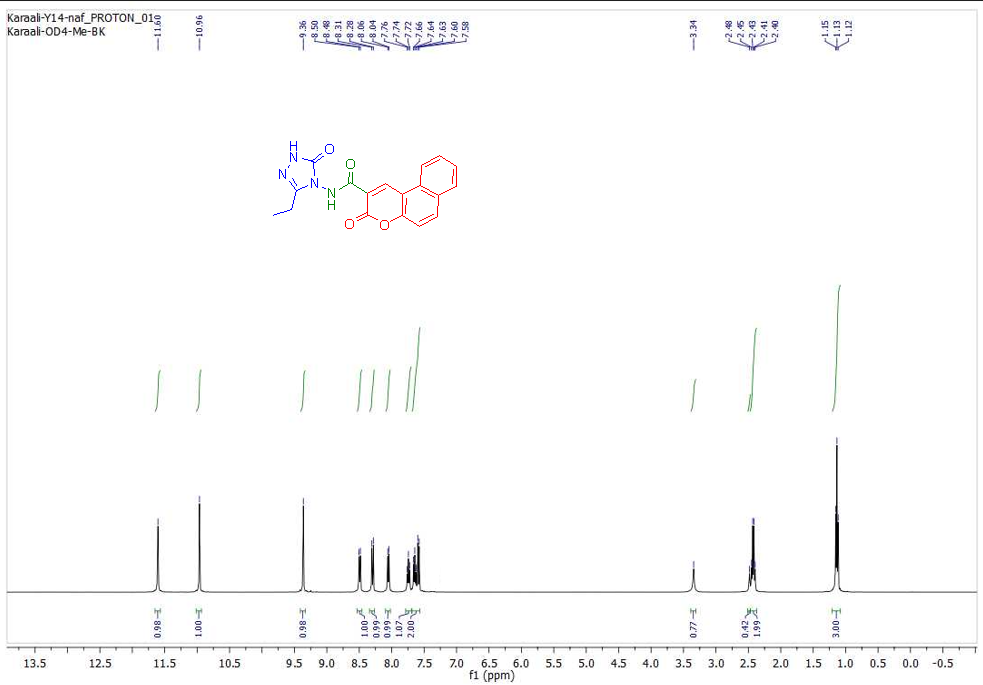


^1^H-NMR spectra of compound **5b**

**
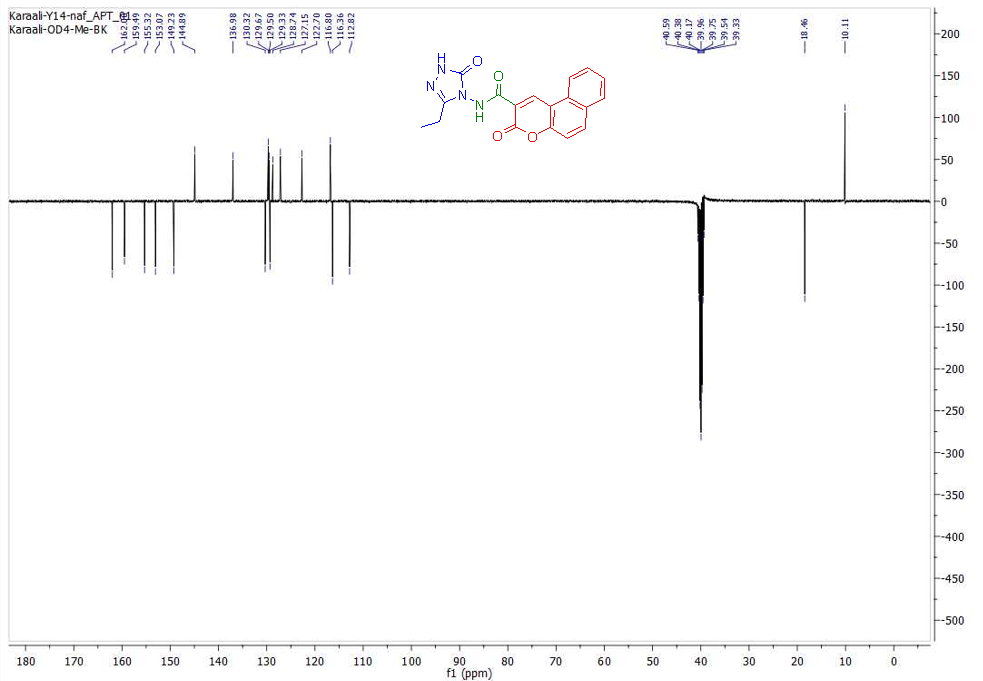
**

^13^C-NMR spectra of compound **5b**

**
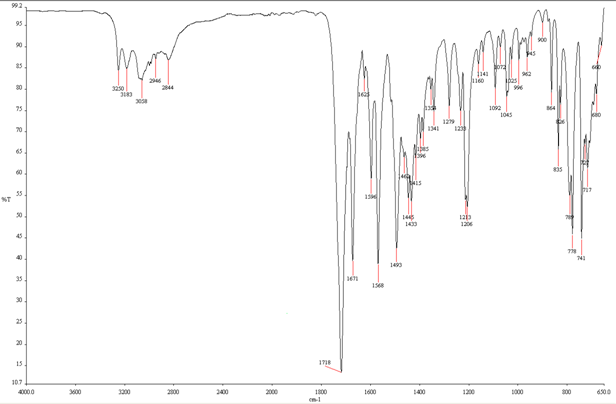
**

IR spectra of compound **5b**

**
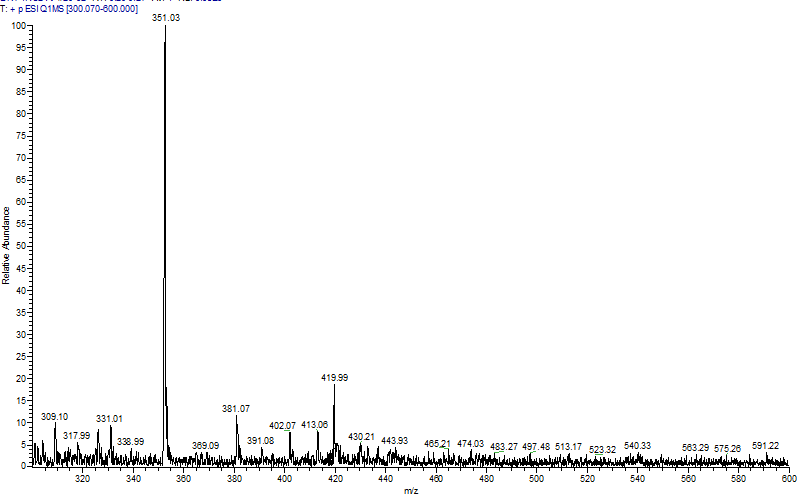
**

MS spectra of compound **5b**

**
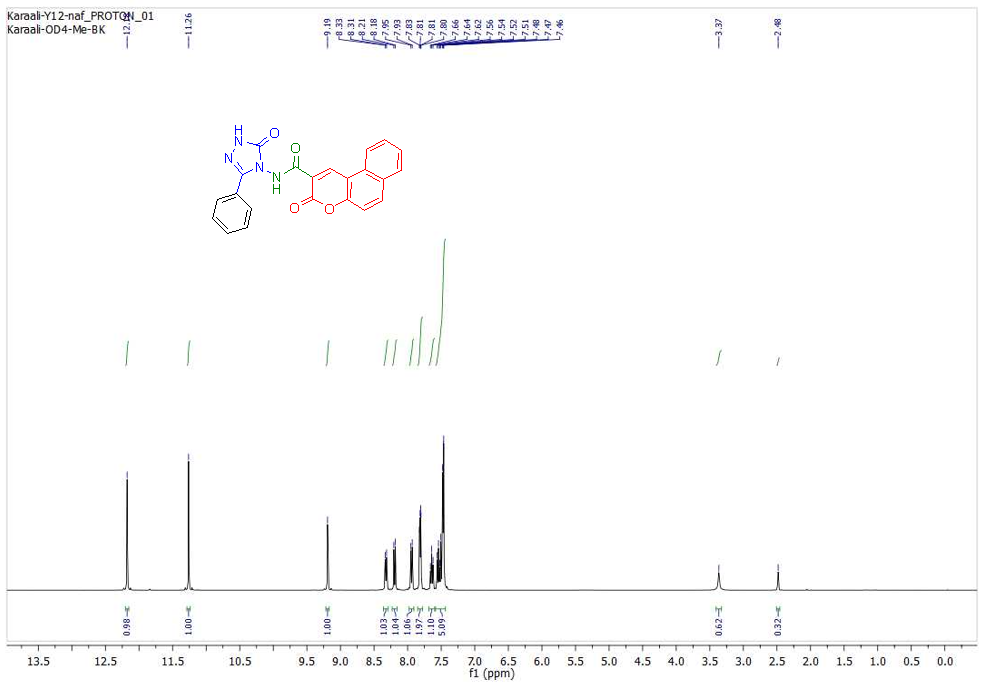
**

^1^H-NMR spectra of compound **5c**


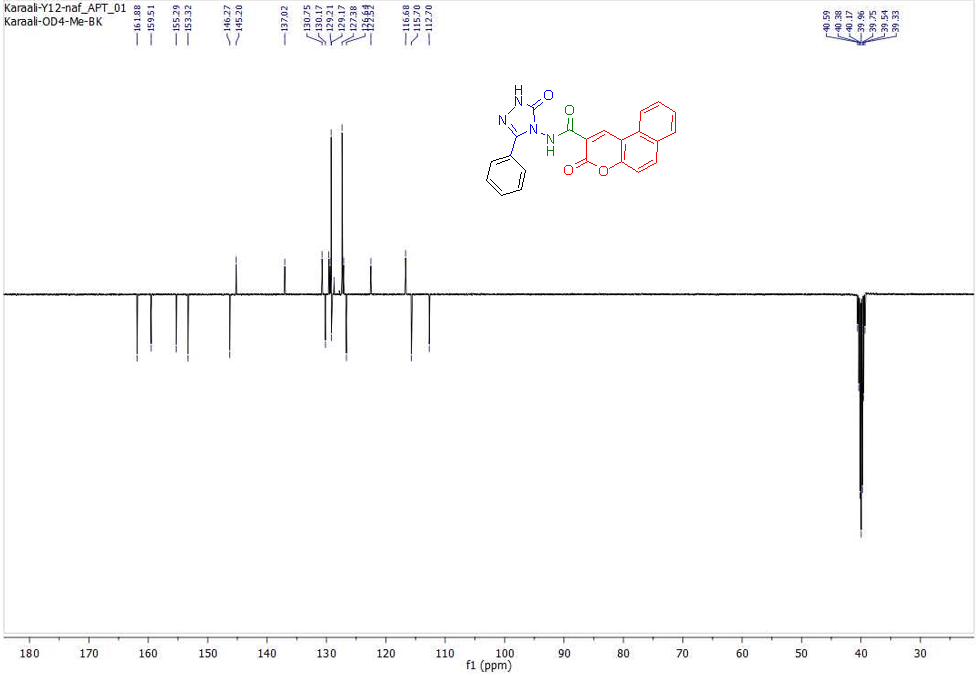


^13^C-NMR spectra of compound **5c**

**
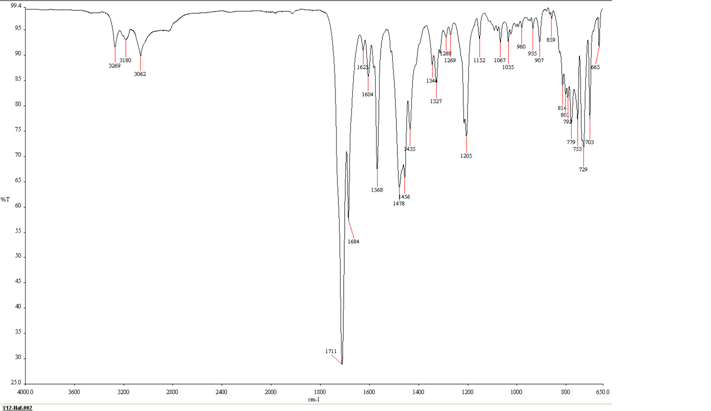
**

IR spectra of compound **5c**

**
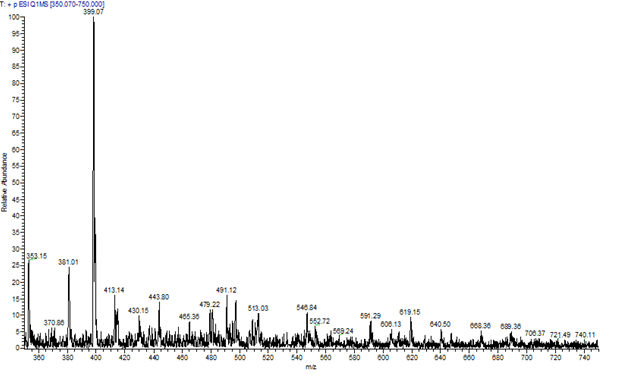
**

MS spectra of compound **5c**

**
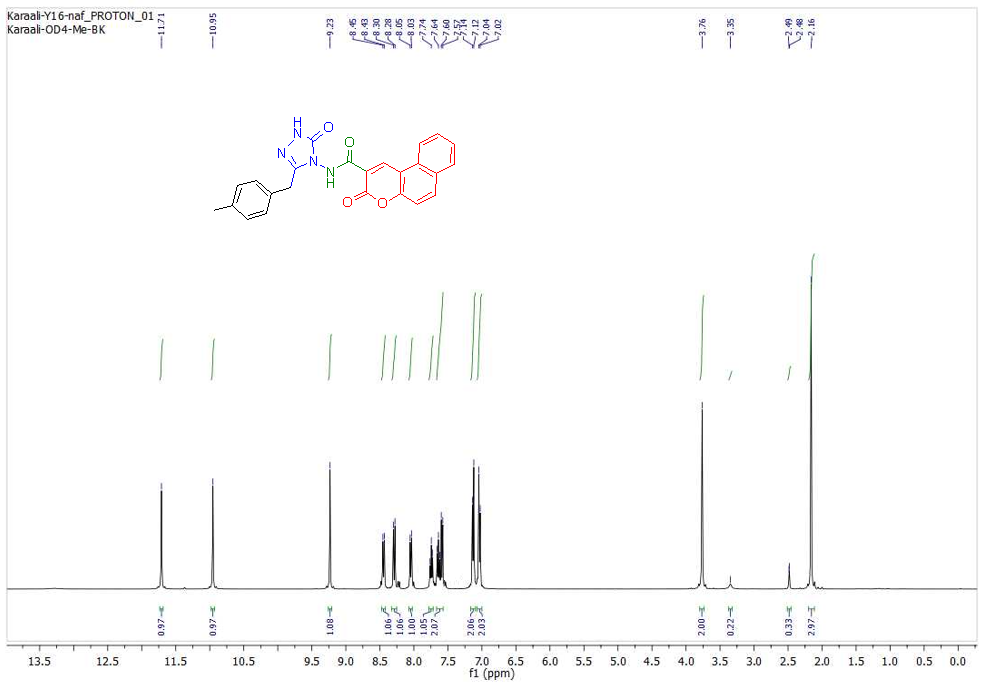
**

^1^H-NMR spectra of compound **5e**

**
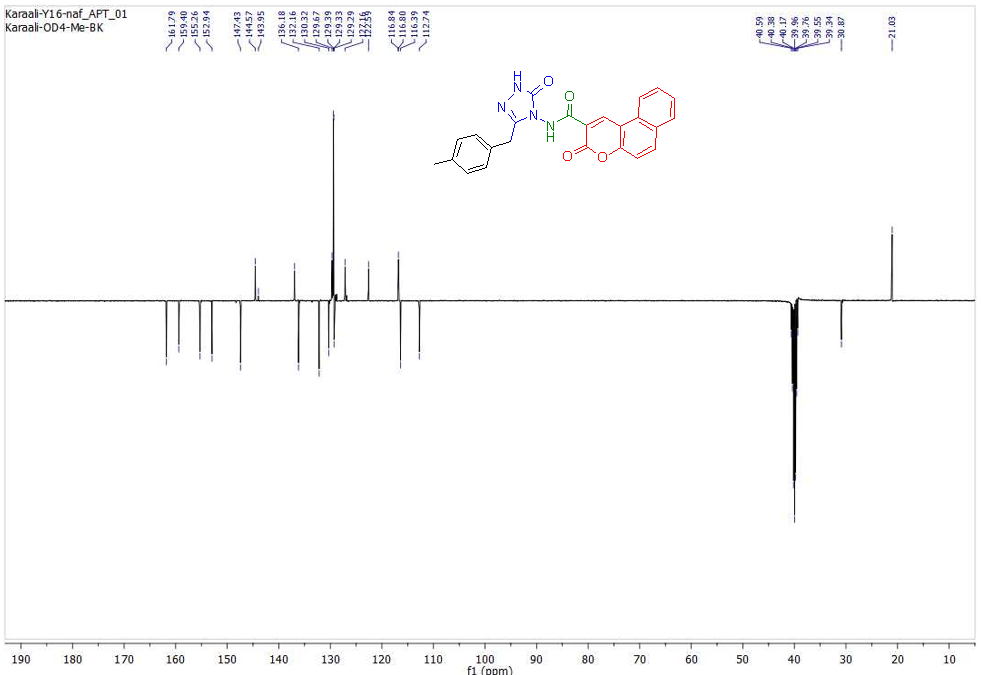
**

^13^C-NMR spectra of compound **5e**

**
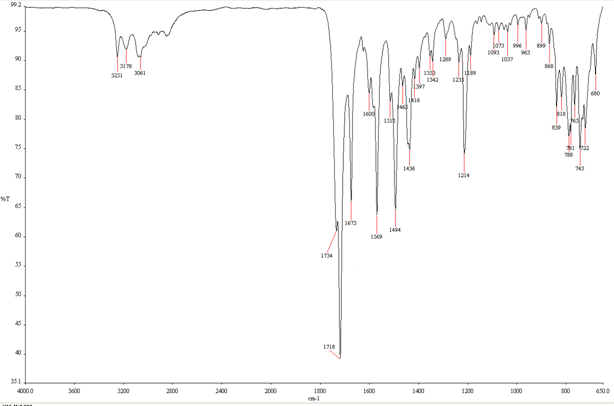
**

IR spectra of compound **5e**

**
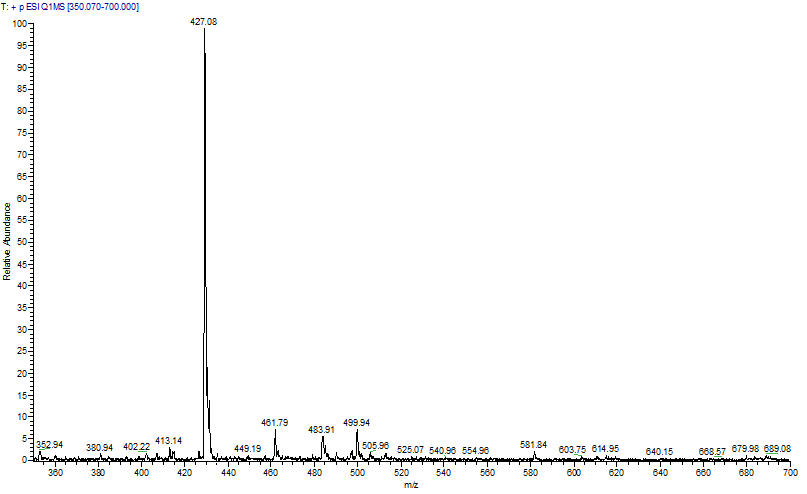
**

MS spectra of compound **5e**

**
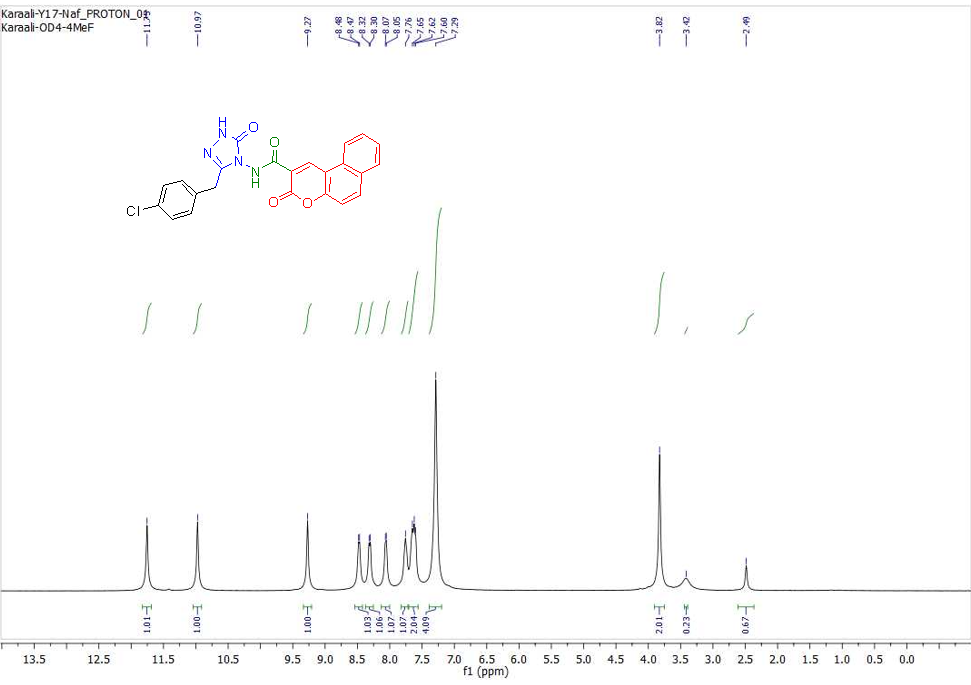
**

^1^H-NMR spectra of compound **5f**


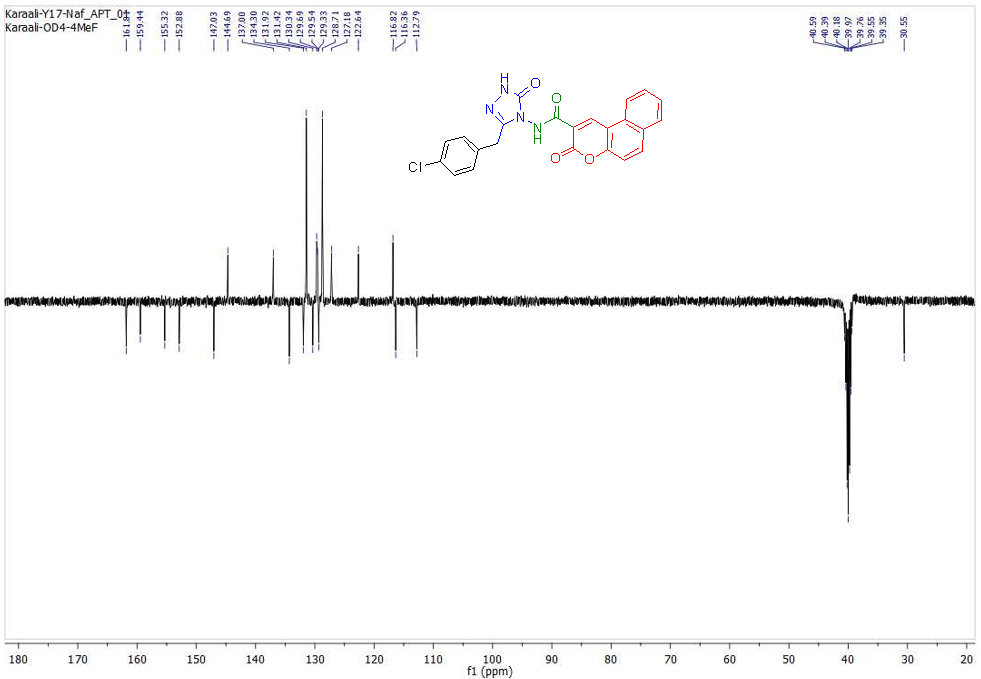


^13^C-NMR spectra of compound **5f**

**
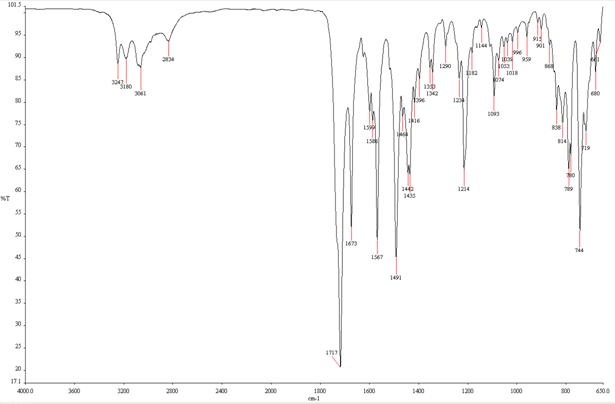
**

IR spectra of compound **5f**

**
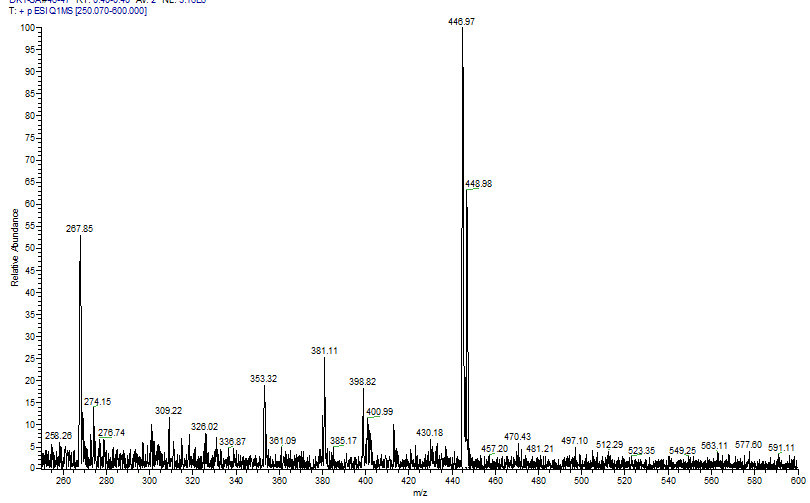
**

MS spectra of compound **5f**
